# Childhood exposure to second-hand smoke (SHS) and risk of breast cancer in postmenopausal never smokers: the Multiethnic Cohort (MEC) study

**DOI:** 10.1186/s13058-025-02202-7

**Published:** 2025-12-25

**Authors:** Inger T. Gram, Song-Yi Park, Lynne R. Wilkens, Christopher A. Haiman, Anna H. Wu, Loïc Le Marchand

**Affiliations:** 1 Department of Community Medicine, Faculty of Health Sciences, UiT The Arctic University of Norway, Tromsø, Norway; 2 Norwegian Centre for E-health Research, University Hospital of North Norway, Tromsø, Norway; 3 Population Sciences in the Pacific Program, University of Hawai’i Cancer Center, Honolulu, HI USA; 4 Department of Population and Public Health Sciences, Keck School of Medicine, University of Southern California, Los Angeles, CA USA

**Keywords:** Breast cancer prevention, Breast cancer risk, Childhood exposure, Cohort studies, Multiethnic Cohort, Passive smoking, Postmenopausal breast cancer, Prospective study, Second-hand smoke

## Abstract

**Background:**

Neither active smoking nor second-hand smoke (SHS) are established causes for breast cancer. We examined the association between daily SHS exposure at home during childhood and postmenopausal breast cancer risk among never smokers in the Multiethnic Cohort (MEC) Study.

**Methods:**

We analyzed data from 24,261 never-smoking postmenopausal female MEC participants, aged 60–89 years in 2008–2012, when they provided information on SHS exposure during childhood in the forth questionnaire (QX4). We identified invasive breast cancer cases and tumour receptor status via linkage to the Hawaii and California Surveillance, Epidemiology and End Results Program cancer registries through December 2019. We used Cox proportional hazards models to estimate age-adjusted hazard ratios (HRs) and 95% confidence intervals (CIs), adjusting for race and ethnicity.

**Results:**

During a mean follow-up of 7.8 years, we identified 709 incident, invasive breast cancer cases. Women reporting any childhood SHS exposure had a 17% higher risk (HR = 1.17, 95% CI: 1.00–1.36) of breast cancer compared with those reporting no childhood exposure, after adjustment for age at QX4 and race and ethnicity. We found higher HRs for breast cancer for those exposed ≥ 3 h/day (HR = 1.24, 95% CI: 1.02–1.51), those exposed ≥ 18 years (HR = 1.26, 95% CI: 1.04–1.53), and those with the highest joint exposure (≥ 3 h/day & ≥18 years) (HR = 1.28, 95% CI: 1.00–1.63). A trend test, with increasing integers from those not exposed to those most exposed, showed a dose response relationship between duration and breast cancer risk for all three associations (all *P*_trend_ ≤0.03). The childhood SHS exposure and breast cancer risk association did not differ, (all *P*_heterogeneity_ ≥0.17), when we stratified on nine established breast cancer risk factors (year of birth, age at QX4, race and ethnicity, education years, age at menarche, parity status, BMI -, physical activity -, and alcohol consumption all at QX4).

**Conclusions:**

Our findings, in this diverse population, add prospective data that daily SHS exposure during childhood may increase the risk of postmenopausal breast cancer. Our results provide additional arguments to establish SHS as a cause of breast cancer and to implement smoking bans in public and private places, when children are present.

**Supplementary Information:**

The online version contains supplementary material available at 10.1186/s13058-025-02202-7.

## Background

 In 2022, breast cancer was the most common cancer globally among women. The highest rates were in France, Australia/New Zealand, Northern America and Northern Europe. However, rapid increases in breast cancer incidence are seen in countries in South America, Africa and Asia as well as in high-income Asian countries [1]. Global age-standardized smoking prevalence was estimated to be 28.5% among males and < 6% among females in 2022. However, the number of smokers remains large because of population growth, particularly in developing countries [2]. About 80% of the world’s smokers live in low- and middle-income countries [3], where children and women are disproportionally exposed to second-hand smoke (SHS), also referred to as environmental, involuntary, passive smoke or tobacco smoke pollution. In 2019, the estimated overall SHS exposure ranged from 12 to 58%, between countries, but with huge variations within many countries [4]. In 2004, the WHO estimated that overall, 40% of children worldwide were exposed to SHS on a daily basis [5].

Neither active smoking nor SHS are established risk factors for breast cancer. Four authoritative reports, published between 2005 and 2014 [6–9], conclude that there is sufficient mechanistic evidence for SHS to cause breast cancer. The two earlier reports [6, 7] concluded that SHS smoking is a causative factor, while the two latter reports [8, 9] classified SHS as *possibly* (class 2B) causative [8] and *suggestive* [9] for causing breast cancer. The relationships of active smoking and SHS as causes of breast cancer are on the IARC evaluation list for the second part of the 2025–2029 period [10].

We previously examined the role of active smoking and breast cancer risk among postmenopausal women in the Multiethnic Cohort (MEC) Study [11, 12]. We found that active smoking before first childbirth increases the risk of postmenopausal breast cancer, to the same extent among drinkers and in non-drinkers of alcohol [11]. Furthermore, the association was comparable across the five major racial and ethnic groups (African American, Japanese American, Latino, Native Hawaiian and White women of the MEC and by hormone receptor status in the postmenopausal women [12]. Our recent report from the Norwegian Women and Cancer cohort study with more than 2,000 breast cancer cases among never smokers found an overall 11% increased breast cancer risk for women reporting childhood SHS exposure from parents compared with those who did not. The corresponding figure confined to postmenopausal women was a 22% statistically significantly higher risk of breast cancer [13].

The purpose of this study was to examine prospectively the association between daily SHS exposure at home during childhood and the risk of breast cancer among postmenopausal never-smoking women.

## Methods

### Study population

The MEC Study consists of more than 215,000 men and women who were aged 45–75 years and living in California and Hawaii at time of cohort entry. The majority of the cohort members belong to one of five racial/ethnic populations: African Americans, Japanese Americans, Latinos, Native Hawaiians, and whites. The cohort has been previously described in detail [14]. Briefly, between 1993 and 1996, participants enrolled in the study by completing a 26-page mailed questionnaire which asked for detailed information about demographic factors, dietary habits, other lifestyle factors, prior medical conditions and family history of common cancers. We identified potential participants through driver’s license files from the state Department of Motor Vehicles, voter registration lists and Health Care Financing Administration (Medicare) data files. The institutional review boards of the University of Hawaii and the University of Southern California approved the study protocol. Informed consent to participate was considered by our IRBs to be implied by the return of the completed baseline questionnaire.

Follow-up questionnaires were mailed to all MEC participants about every five years to update select exposures or assess new exposures. The fourth questionnaire (QX4), that was administered in 2008–2012, included the question: “Were you exposed to smoke from other people’s cigarette smoking or tobacco products on a *daily* basis?” The participant could answer (yes/no) to four settings for exposure:

(1) Childhood at home; (2) Adulthood at home; (3) At work or in social settings such as restaurants, bars, bowling alleys, bingo halls, and friends’ home; (4) Currently exposed to the smoke of others? If the answer to the question was yes, the questionnaire asked for how many *hours per day*? and for *how many years*? Our primary exposure of interest is SHS exposure during childhood, as children are the least able to protect themselves from SHS.

In this report, we used information on year of birth, years of education, age at menarche, number of children, race and ethnicity, and height from the baseline questionnaire at which time all women were postmenopausal. We used updated information from QX4 including age at QX4, smoking status, current weight for calculating body mass index (BMI, kg/m^2^), physical activity, and current alcohol consumption. Altogether, 53,619 postmenopausal women completed the QX4. We excluded women who did not belong to one of the five targeted racial and ethnic groups (*n* = 3,242), had a prior breast cancer (*n* = 4,661), had missing information on active smoking status (*n* = 1,998), and SHS during childhood at home (*n* = 895), were current or former smokers at QX4 (*n* = 17,575), and were outside the age range of 60–89 years or older at QX4 (*n* = 850). As a result, 24,261 postmenopausal women aged 60–89 years, who were never smokers, remained for the main analysis.

We identified invasive incident cancer cases by linkage to the Surveillance, Epidemiology, and End Results (SEER) Program cancer registries covering Hawaii and California. We classified breast cancer cases according to the organ site code (C50) in the International Classification of Diseases, Tenth Revision and according to oestrogen (ER), progesterone (PR) and Human Epidermal Growth Factor Receptor 2 (HER2) tumour receptor status categories [ER-positive (ER+), ER-negative (ER–), PR+, PR–, HER2+, HER2–] based on information from the SEER registries. We identified deaths by linkage to death certificate files in Hawaii and California and to the National Death Index. Case ascertainment and vital status were completed through December 31, 2019. We calculated person-years from the start of follow-up (2008–2012 of QX4 response) to the date of invasive breast cancer diagnosis, death or the end of follow-up, whichever occurred first.

### Statistical analysis

For the distribution of selected characteristics of the study population, we calculated percentages (%) or means with standard deviations. We calculated breast cancer incidence rates per 100 000 women, age standardized to the 2000 US standard population [15] and left truncated at age 60. Observation period was from QX4 to the first event of invasive breast cancer diagnosis, death, or censor date of 2019. We used Cox proportional hazards regression to model time to breast cancer, with age as the underlying time scale to estimate hazard ratios (HRs) with 95% confidence intervals (CIs) for the associations with SHS exposure. We included the five main race and ethnicity groups as a strata variable when applicable, and age at QX4 as a covariate. The proportional hazards assumption was tested using Schoenfeld residuals and there was no evidence of violation [16, 17].

We estimated the overall association for women reporting SHS exposure daily during childhood at home compared with those reporting no such exposure. We explored other parameterizations of SHS childhood exposure, as levels of SHS exposure at home during childhood based on exposure duration in hours (< 3/3 + hours/day), in years (< 18/18 + years), and by the four joint levels of exposure (< 3 h/day & <18 years, ≥ 3 h/day & <18 years, < 3 h/day & ≥18 years, ≥ 3 h/day & ≥18 years). We conducted tests for linear trends by including an ordinal exposure variable with equally spaced scores in models with three categories for duration (only hours and only years) and five categories for the joint duration (hours and years) of SHS childhood exposure given above, with the common reference group as the first category.

We performed competing risk analysis using cause-specific models for time to breast cancer hormone receptor status outcomes (ER + and ER–, PR + and PR–, HER2 + and HER2–), with censoring at diagnosis for any breast cancer cases with an unknown receptor status [17–19]. To compare the parameters by tumour receptor status, we implemented an augmented data approach as described in Lunn and McNeil [20]. This approach computes simultaneous models for breast cancer of each receptor status type. Heterogeneity by tumour receptor status categories was assessed by a Wald test comparing the interaction between tumour receptor event type and SHS exposure, using robust variance estimates [16].

We tested for heterogeneity in the SHS exposure and breast cancer association by nine risk factors [year of birth (< 1930, 1930–1939, ≥ 1940), age at QX4 (60–69,70–79,80–89), race and ethnicity (African American, Japanese American, Latino, Native Hawaiian, White), education years (≤ 12, > 12), age at menarche (≤ 12, > 12), parity status (nulliparous, parous), BMI at QX4 (< 25, ≥ 25 kg/m^2^), physical activity at QX4 (< 45, ≥ 45 min/day), alcohol consumption at QX4 (nondrinkers, drinkers)], using the Wald test for interaction terms, adjusting for age at QX4 and race/ethnicity when applicable.

We performed additional multivariable adjusted analyses for the main association between SHS and breast cancer overall and by tumor subtypes using (1) a complete-case approach, (2) a separate category for missing values and (3) with imputed missing values.

Furthermore, we calculated HRs with 95% CIs for the associations with SHS exposure daily during adulthood at home [yes, no], at work or in social settings [yes, no], or any current exposure [yes, no]. We performed analyses in the subset of women who answered ‘yes’ to both SHS exposure daily during childhood and adulthood at home compared to those reporting ‘no’ to both. Likewise, we performed analyses in the subset of women who answered ‘yes’ to the three SHS exposures (daily during childhood and adulthood at home and to SHS exposure at work) compared with those reporting ‘no’ to all three exposures, and the subset of women who answered ‘yes’ to the four SHS exposures (daily during childhood and adulthood at home and to SHS exposure at work, and currently any exposure) compared with those reporting ‘no’ to all four.

Moreover, we re-analyzed the childhood exposure and breast cancer association, overall, and according to the joint duration of exposure, testing for trend, and according to the five race and ethnicity groups, testing for heterogeneity. For these analyses, the reference group was those not exposed to any SHS.

Finally, to see if the SHS - breast cancer association diminishes the longer the women have been postmenopausal we reanalyzed the main data adjusting for age at QX4 and race/ethnicity, but excluding women aged ≥ 80 years. We performed the analyses using SAS version 9.4 (SAS Institute Inc., Cary, NC).

## Results

Altogether, 39.2% (n *=* 9,509) of the 24,261 eligible women, reported SHS exposure daily, during childhood at home. We identified 709 incident, invasive breast cancer cases, during a mean follow-up of 7.8 years. The truncated age-adjusted incidence rate for breast cancer overall was 330.1 per 100 000 person-years. Japanese American and Native Hawaiian women constituted the largest (35.5%) and the smallest (6.1%) proportion of the study population, respectively. Women who reported childhood SHS exposure were younger at QX4, at breast cancer diagnosis, and at menarche, and were more likely to have more education, have no children, have fewer children, have a higher BMI, be more physically active, and be alcohol consumers at QX4, compared with those not reporting childhood SHS exposure (all *Ps* < 0.001) (Table 1).

**Table 1:** Selected characteristics of never smokers, overall and by exposure to second-hand smoke at home during childhood, the Multiethnic Cohort study

Characteristics at cohort entry	Never smokers	Second-hand smoke exposure		*P*-value^a^
	Total	No	Yes	
No. of participants	24,261	14,752	9,509	
Age at QX4, mean (SD)	74.1 (7.8)	75.1 (7.7)	72.7 (7.6)	< 0.001
Age at diagnosis, mean (SD)	77.1 (7.7)	78.2 (7.8)	75.8 (7.3)	< 0.001
Years of follow-up, mean (SD)	7.8 (2.1)	7.8 (2.2)	8.0 (2.0)	< 0.001
No. of breast cancer cases (%)	709 (2.9)	392 (2.7)	317 (3.3)	0.002
Incidence/100,000 (95% CI)^b^	330.1 (293.1-367.2)	291.7 (246.2-337.3)	375.1 (316.0-434.1)	
Race and ethnicity, n (%)				
African American	2998 (12.4)	2012 (13.6)	986 (10.4)	< 0.001
Japanese American	8621 (35.5)	5285 (35.8)	3336 (35.1)	
Latino	5693 (23.5)	4038 (27.4)	1655 (17.4)	
Native Hawaiian	1475 (6.1)	852 (5.8)	623 (6.6)	
White	5474 (22.6)	2565 (17.4)	2909 (30.6)	
Family history of breast cancer, n (%)	2446 (10.6)	1448 (10.4)	998 (10.9)	0.21
> 12 years of education, n (%)	14,827 (61.7)	8336 (57.1)	6491 (68.8)	< 0.001
Age at menarche, mean (SD)	13.1 (1.7)	13.1 (1.7)	13.0 (1.7)	< 0.001
Age at natural menopause, mean (SD)	49.2 (4.9)	49.2 (4.9)	49.1 (4.9)	0.50
Parous women, n (%)	21,184 (87.5)	13,010 (88.4)	8174 (86.1)	< 0.001
Number of children, mean (SD)^c^	3.1 (1.6)	3.2 (1.6)	3.0 (1.5)	< 0.001
Age at first childbirth, mean (SD)^c^	23.9 (4.6)	23.8 (4.6)	23.9 (4.5)	0.18
Body mass index at QX4 (kg/m^2^), mean (SD)	25.8 (5.5)	25.5 (5.4)	26.1 (5.7)	< 0.001
Physical activity at QX4 (h/day), mean (SD)^d^	1.21 (1.29)	1.17 (1.28)	1.27 (1.29)	< 0.001
Nondrinkers at QX4, n (%)	17,837 (74.7)	11,521 (79.4)	6316 (67.4)	< 0.001

QX4, fourth questionnaire

^a^T-test or chi-square test for differences between exposed and nonexposed groups

^b^Rates were age-standardized to the 2000 US standard population and left truncated at age 60 years

^c^Among parous women

^d^Moderate to vigorous activities

Supplementary Table 1 shows that the ethnic-specific truncated age-standardized incidence rates per 100 000 women for breast cancer ranged from 669.9 among African American women reporting childhood SHS exposure to 219.8 among Latina women reporting no such exposure (Supplementary Table 1).

More than half of the White women (53.1%) reported childhood SHS exposure, while the corresponding figures for the other groups were lower: Native Hawaiian (42.2%), Japanese American (38.7%), African American (32.9%), and Latino (29.1%) women.

Compared with women reporting no childhood exposure, those reporting childhood SHS exposure at home had a 17% higher risk (HR = 1.17, 95% CI: 1.00–1.36) of breast cancer, after adjustment for age at QX4 and race and ethnicity. (Table 2) We found higher HRs for breast cancer for those exposed ≥ 3 h/day (HR = 1.24, 95% CI: 1.02–1.51), those exposed ≥ 18 years (HR = 1.26, 95% CI: 1.04–1.53), and those with the highest joint exposure (≥ 3 h/day & ≥18 years) (HR = 1.28, 95% CI: 1.00–1.63). A trend test, with increasing integers from those not exposed to those most exposed, showed a dose response relationship between duration and breast cancer risk for all three associations (all *P*_trend_ ≤0.03). (Table 2)

**Table 2:** Second-hand smoke (SHS) at home during childhood and breast cancer risk by exposed hours per day and exposed years, the Multiethnic Cohort Study, 2008–2019

SHS exposure	No. of participants	No. of cases	HR (95% CI)^a^
No^b^	14,752	392	1.00 (ref)
Yes	9509	317	1.17 (1.00-1.36)
Among exposed			
Exposed hours			
< 3 h/day	3575	116	1.11 (0.90–1.37)
≥ 3 h/day	4159	148	1.24 (1.02–1.51)
P_trend_^c^			0.03
Exposed years			
< 18 years	4385	137	1.10 (0.90–1.34)
≥ 18 years	4033	148	1.26 (1.04–1.53)
P_trend_^c^			0.02
Exposed hours and years			
< 3 h/day & <18 years	2090	62	1.02 (0.78–1.33)
≥ 3 h/day & <18 years	1710	57	1.18 (0.89–1.56)
< 3 h/day & ≥18 years	1259	46	1.23 (0.90–1.67)
≥ 3 h/day & ≥18 years	2274	85	1.28 (1.00-1.63)
P_trend_^d^			0.02

^a^Adjusted for age at QX4 and race/ethnicity

^b^Common reference group

^c^Trend variable assigned consecutive numbers for the three categories including the common reference group

^d^Trend variable assigned consecutive numbers for the five categories including the common reference group

Table 3 shows that women reporting childhood SHS exposure had HR estimates above 1, compared with the reference group, for all the six receptor status subgroups. The strongest associations for SHS exposure were found for women with HER2+ (HR = 1.93, 95% CI: 1.15–3.22), PR- (HR = 1.57, 95% CI: 1.13–2.18), and ER-(HR = 1.50, 95% CI: 0.96–2.34) tumors. We found no evidence of statistical difference in the association by ER status [(610 ER + vs. e 81 ER– ) (*P*_heterogeneity_ = 0.24)], but there was evidence of difference in the association by PR status [(539 PR + vs. 149 PR–) (*P*_heterogeneity_ = 0.04)] and by HER2 status [(62 HER2 + vs. 611 HER2–) (*P*_heterogeneity_ = 0.04)].

**Table 3:** Second-hand smoke (SHS) at home during childhood and breast cancer risk, the Multiethnic Cohort Study, 2008–2019

SHS Exposure	ER-positive		ER-negative		PR-positive		PR-negative		HER2-postivie		HER2-negative	
	No. of cases	HR (95% CI)^a^	No. of cases	HR (95% CI)^a^	No. of cases	HR (95% CI)^a^	No. of cases	HR (95% CI)^a^	No. of cases	HR (95% CI)^a^	No. of cases	HR (95% CI)^a^
No	341	1.00 (ref)	41	1.00 (ref)	306	1.00 (ref)	74	1.00 (ref)	27	1.00 (ref)	342	1.00 (ref)
Yes	269	1.12 (0.95–1.32)	40	1.50 (0.96–2.34)	233	1.07 (0.90–1.27)	75	1.57 (1.13–2.18)	35	1.93 (1.15–3.22)	269	1.12 (0.95–1.32)
P_heterogeneity_^b^	0.24				0.04				0.04			

^a^Adjusted for age at QX4 and race/ethnicity

^b^Based on a competing risk model

Table 4 shows that breast cancer risk of the youngest women born (i.e. born in 1940 or later) was 36% higher (HR = 1.36, 95% CI: 1.08–1.71) and women with age at menarche > 12 years was 28% higher (HR = 1.28, 95% CI: 1.02–1.62) for daily childhood SHS exposure compared to no such exposure. The childhood SHS exposure and breast cancer risk associations did not differ statistically significantly when we stratified on the nine established breast cancer risk factors, including race and ethnicity, displayed in the table (all *P*_heterogeneity_ ≥0.17).

**Table 4:** Second-hand smoke (SHS) at home during childhood and breast cancer risk by nine risk factor subgroups, the Multiethnic Cohort Study, 2008–2019

Subgroup	SHS exposure	No. of participants	No. of cases	HR (95% CI)^a^	*P*_heterogeneity_
Year of birth					
< 1930	No	4106	95	1.00 (ref)	
	Yes	1738	42	1.01 (0.70–1.45)	
1930–1939	No	5743	163	1.00 (ref)	
	Yes	3280	103	1.05 (0.81–1.34)	
≥ 1940	No	4903	134	1.00 (ref)	
	Yes	4491	172	1.36 (1.08–1.71)	0.17
Age at QX4					
60–69 years	No	4170	120	1.00 (ref)	
	Yes	3921	154	1.34 (1.06–1.71)	
70–79 years	No	5693	156	1.00 (ref)	
	Yes	3469	114	1.15 (0.90–1.47)	
80–89 years	No	4889	116	1.00 (ref)	
	Yes	2119	49	0.93 (0.66–1.30)	0.20
Race and ethnicity					
African American	No	2012	47	1.00 (ref)	
	Yes	986	36	1.43 (0.92–2.23)	
Japanese American	No	5285	161	1.00 (ref)	
	Yes	3336	126	1.18 (0.94–1.50)	
Latino	No	4038	74	1.00 (ref)	
	Yes	1655	40	1.29 (0.88–1.90)	
Native Hawaiian	No	852	40	1.00 (ref)	
	Yes	623	27	0.88 (0.54–1.45)	
White	No	2565	70	1.00 (ref)	
	Yes	2909	88	1.08 (0.79–1.49)	0.56
Education					
≤ 12 years	No	6257	126	1.00 (ref)	
	Yes	2941	78	1.21 (0.90–1.61)	
> 12 years	No	8336	263	1.00 (ref)	
	Yes	6491	235	1.12 (0.93–1.33)	0.57
Age at menarche					
≤ 12 years	No	7179	217	1.00 (ref)	
	Yes	5080	177	1.08 (0.88–1.32)	
> 12 years	No	7406	171	1.00 (ref)	
	Yes	4336	137	1.28 (1.02–1.62)	0.26
Parity					
Nulliparous	No	1705	50	1.00 (ref)	
	Yes	1317	50	1.30 (0.87–1.94)	
Parous	No	13,010	342	1.00 (ref)	
	Yes	8174	266	1.14 (0.97–1.35)	0.70
Body mass index at QX4					
< 25 kg/m^2^	No	7284	159	1.00 (ref)	
	Yes	4384	120	1.19 (0.93–1.51)	
≥ 25 kg/m^2^	No	6639	216	1.00 (ref)	
	Yes	4715	185	1.11 (0.91–1.36)	0.66
Physical activity at QX4					
< 45 min/day	No	7382	187	1.00 (ref)	
	Yes	4292	135	1.14 (0.91–1.43)	
≥ 45 min/day	No	7083	202	1.00 (ref)	
	Yes	5092	179	1.18 (0.96–1.45)	0.77
Alcohol consumption at QX4					
Nondrinkers	No	11,521	299	1.00 (ref)	
	Yes	6316	206	1.15 (0.96–1.38)	
Drinkers	No	2986	89	1.00 (ref)	
	Yes	3054	108	1.20 (0.90–1.59)	0.99

QX4, fourth questionnaire

^a^Adjusted for age at QX4 and race/ethnicity

Supplemental Tables 2 and 3 show the results from main Tables 2 and 3 multivariable adjusted analysis using (1) a complete-case approach, (2) a separate category for missing values and (3) with imputed missing values. The results are materially the same, but the associations are somewhat weaker. (Supplementary Tables 2 and 3).

Supplemental Table 4, shows that women exposed to SHS at home during both childhood and adulthood (*n* = 5369) had a 27% higher (HR = 1.27, 95% CI: 1.06–1.54) risk compared to those who reported neither exposure. Women who reported exposure in three settings (during childhood and adulthood and at work/social settings) (*n* = 3928) had a 34% higher (HR = 1.34, 95% CI: 1.08–1.66) risk compared to those who reported no exposure in any of these settings. The 4.8% (*n* = 1155) women who reported SHS exposures in all four settings, had a 34% higher (HR = 1.34, 95% CI: 0.96–1.86) risk compared to the 7, 679 women who reported no such exposures. (Supplementary Table 4).

Among female MEC participants, 34.4% (*n* = 8,334) reported adulthood SHS exposure at home, 41.2% (*n* = 9,997) reported SHS exposure at work/social settings, and 9.4% (*n* = 2,271) reported current SHS exposure in any setting. Among the women reporting no SHS exposure in childhood (*n* = 14,752), constituting our main reference group, 39.5% (*n* = 5,823) reported at least one other SHS exposure. When we excluded women exposed to any SHS from the reference group, the overall association for childhood SHS exposure and breast cancer was strengthened [(HR = 1.25, 95% CI: 1.05–1.49). (*n* = 18,438/ 537 cases)].

**Fig. 1:**
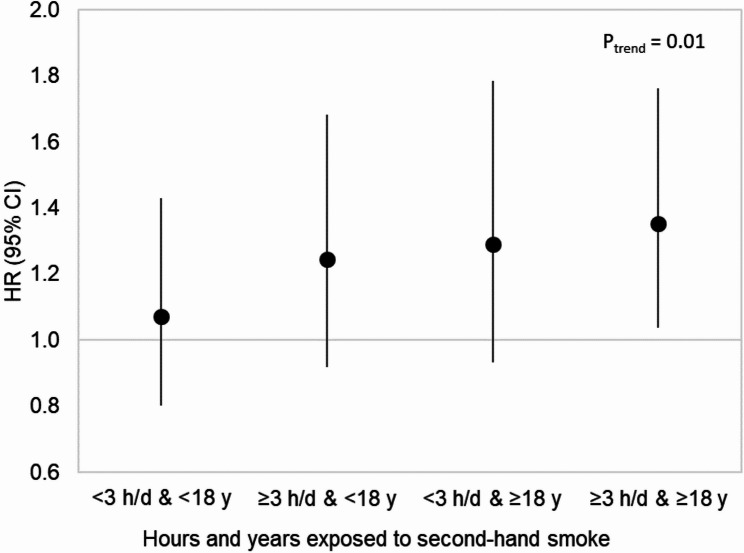
Second-hand smoke (SHS) at home during childhood and breast cancer risk by joint exposure of hours per day and years, the Multiethnic Cohort Study, 2008–2019. The reference group was those not exposed to any SHS (*n* = 8,*929)*. Trend test was based on consecutive numbers assigned to the five categories including the reference group

Figure 1 shows the higher HRs for breast cancer for all four joint associations of duration of childhood SHS exposure, compared with women not reporting any SHS exposure (*n* = 8,929). The association became a little stronger with this clean reference group. A trend test, including the common reference group, showed a dose-response relationship (*P*_trend_ = 0.01).

**Fig. 2:**
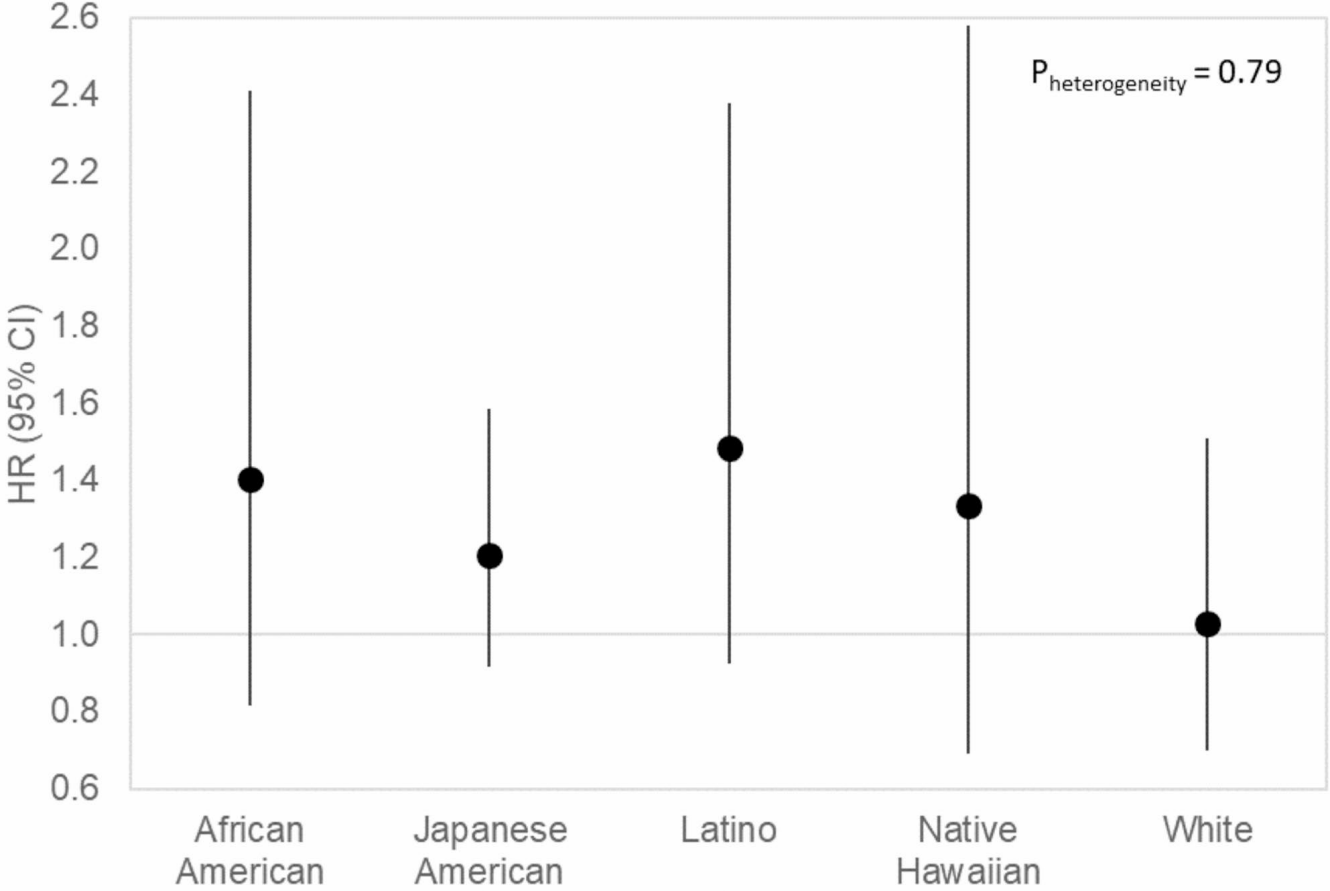
Second-hand smoke (SHS) at home during childhood and breast cancer risk by race and ethnicity, the Multiethnic Cohort Study, 2008–2019. The reference group was those not exposed to any SHS. (*n* = 8,*929)*

Figure 2 shows that all racial and ethnic groups had HRs above 1.0 for the childhood SHS exposure and breast cancer association, compared with women not reporting any SHS exposure (*n* = 8,929). The HR estimates became a bit stronger when this clean reference group was used. All the 95% CI included 1.0 and there was no evidence of heterogeneity in risk association between the five race/ethnicity groups (*P*_heterogeneity_ = 0.79).

Supplemental Tables 5–8 show the study population after excluding women aged ≥ 80 years., i.e. women who have been postmenopausal the longest. These results were largely the same as those shown in Tables 1, 2, 3 and 4. The overall association between SHS exposure and postmenopausal breast cancer, adjusted for age at QX4 and race/ethnicity, became stronger HR 1.24 (95% CI: 1.05–1.47), especially for those with ER positive, PR negative and HER2 positive tumours.

## Discussion

In this racially and ethnically diverse cohort of never smokers, we found a higher risk of breast cancer among postmenopausal women reporting SHS exposure, daily during childhood at home, compared with those reporting no such exposure. Furthermore, there was an exposure response relationship with duration of exposure and breast cancer risk. Both associations were strengthened when we excluded women exposed to any SHS from the reference group. The SHS exposure and breast cancer risk association did not show statistical evidence of heterogeneity when we stratified according to nine breast cancer risk factors, including race and ethnicity. Nonetheless, the youngest women in the MEC experienced an increase in risk of breast cancer that was of a magnitude similar to that of parous ever smokers who had smoked more than 5 years before their first live childbirth, for all four hormone receptor types examined (ER+,ER-;PR+;PR-) in our previous MEC paper [11]. These results among multiethnic and multiracial participants differ from results among Norwegian women [13] which showed little differences in age-adjusted HR estimates for the four (ER + 17%, ER-8%, PR + 10%, PR-16%) hormone tumour receptor types that were investigated.

Since the last authoritative reports published in 2012 (8], and 2014 (9], results from at least 13 cohort studies with > 500 breast cancer cases [11–13, 21–30], and 11 reviews, meta-analysis and pooled analysis [31–40], examining the relationship between smoking (active or SHS or both) and breast cancer risk, have been published.

Four of the 13 cohort studies, published between 2013 and 2017, examined the risk of both SHS and active smoking and breast cancer risk [22, 24, 27, 28]. Three [22, 24, 27, 28]. found a higher risk of breast cancer for both active smoking and never smoking women exposed to different measures of SHS. The Sister study [28], conducted in the USA and Puerto Rico, did not find an association with active smoking and breast cancer. Among never smokers, they found that those exposed to SHS throughout childhood until age 18 years, versus not being exposed had a 17% higher breast cancer risk. Also, women exposed to household smoking in utero versus not being exposed had a 16% higher breast cancer risk [28].

The reviews, meta-analysis and pooled analysis [31–41] examined either; both active smoking and SHS exposure [31, 32, 34, 36, 39], only active smoking [33, 37, 41] or only SHS exposure [35, 38, 40] and breast cancer risk, compared with those not exposed. The reports conclude that active smoking [31–34, 36, 37, 39, 41] and SHS exposure [31, 32, 34–36, 38, 40] most likely increases breast cancer risk. The Japanese pooled analysis of nine population-based studies [39], found a higher breast cancer risk for active smokers, but not for never smokers exposed to SHS. Conversely, the meta-analysis including 53 Chinese studies of which 11 case-control studies concerned breast cancer [38], found that women exposed to SHS had a 57% higher breast cancer risk compared with those not exposed.

It has been hypothesized [6, 7, 31] that SHS exposure may have a greater effect on the risk of breast cancer among pre- than post-menopausal women. The two [36, 40] meta-analyses that stratified by menopausal status for the association between SHS exposure and breast cancer both found similar excess risks, that was higher in pre- compared with post-menopausal women. In the study by He et al. [36], based on 11 studies, only the association with premenopausal breast cancer achieved statistical significance. The study by Possenti et al. [40], based on 27 studies, showed RR estimates that were statistically significant in both strata of menopausal status. Neither of the two studies [36, 40] found evidence of statistical difference by menopausal status.

According to a 2008 report, the evidence in 2005 from epidemiological studies on SHS and risk of premenopausal breast cancer was stronger than the evidence for the association between SHS and lung cancer established in 1986 [42]. This conclusion is fully supported by two recent meta-analysis conducted by Possenti et al. [40, 43]. The authors showed that compared with those not exposed, those reporting overall SHS exposure had a 24% higher risk for breast cancer [40], and a 24% higher risk for lung cancer [43]. The meta-analysis included 52 studies on breast cancer [40], and 82 studies on lung cancer [43].

One major strength of our study is that we have a population of never smokers with information on duration of daily childhood SHS exposure. Our estimates are consistent with showing a positive association between SHS exposure daily during childhood and postmenopausal breast cancer risk. Other strengths are that we could address the association of interest by six tumour receptor status classes, according to nine established breast cancer risk factors in a diverse study population derived from the general population. Also, our study has > 500 breast cancer cases which are usually the criteria for being included in meta- and pooled analyses. Such overall analysis is essential to establish a causal relationship when the association is weak, the exposure is self-reported and has not been measured in a consistent way.

Furthermore, our results remained materially the same with slight weakening of the associations obtained when we performed the three different multivariable adjusted analysis. The possibility of over-adjustment exists as it is unlikely that childhood SHS exposure would be causally linked with being more physically active, having more education and fewer children, as a few examples. Another strength is that we explored whether the effect of daily childhood SHS exposure on breast cancer risk diminish with increasing duration since menopause. When we excluded the women that had been postmenopausal the longest, the overall association between daily childhood SHS exposure and postmenopausal breast cancer became stronger, but the general picture was materially the same.

The main limitation of the present analysis is the short follow-up time resulting in around 700 incident breast cancer cases among never smokers. This relatively small number of cases resulted in unstable estimates for the subgroup analyses. Our stratified analyses by different hormone receptor tumor types are likely underpowered. Furthermore, the women recruited in the MEC were born between 1918 and 1948, and 20.5% had died prior to QX4 mailings. We excluded women aged 90 + at the time of the QX4, to minimize the bias due to selection of healthy women. There may be residual confounding due to the self-reported SHS exposure and unmeasured confounding factors.

In 2002, Hecht described that among the over 60 known carcinogens in tobacco smoke, more than twenty had been identified to induce mammary tumours in rodents. These compounds were also found in breast tissue of female smokers [44].

In 2022, Li and Hecht updated the number of carcinogens to be 83, i.e. 37 found in unburned tobacco and 80 in mainstream tobacco smoke [45]. SHS is comprised of mostly side stream smoke (85%) and some exhaled mainstream smoke (15%), with an estimate of at least 7,000 chemicals, many which are toxic. Indoor SHS is expected to persist for hours and become more toxic with time [46]. According to the 2021 global progress report on implementation of the WHO Framework Convention on Tobacco Control, 171 (94%) of 181 countries have banned smoking in public places. However, as of 2020, only 37 (20%) have banned smoking in private vehicles when children are present [47].

In conclusion, our findings, in this diverse population, add prospective data that daily SHS exposure during childhood may increase the risk of postmenopausal breast cancer. Our results provide additional arguments to establish SHS as a cause of breast cancer and to implement smoking bans in public and private places, when children are present.

## Supplementary Information

Below is the link to the electronic supplementary material.

Supplementary Material 1

## Data Availability

For information on how to gain access to data from the Multiethnic Cohort, please see: [https://www.uhcancercenter.org/for-researchers/mec-data-sharing] (https:/www.uhcancercenter.org/for-researchers/mec-data-sharing).

## Abbreviations

BMI: body mass index
CI: confidence interval
ER+: oestrogen receptor positive
ER–: oestrogen receptor negative
HER2+: human epidermal growth factor receptor 2 positive
HER2‒: human epidermal growth factor receptor 2 negative
HR: hazard ratio
MEC: Multiethnic Cohort
PR+: progesterone receptor positive
PR–: progesterone receptor negative
SD: standard deviation
SHS: second-hand smoke (i.e. environmental, involuntary, passive smoke or tobacco smoke pollution.)
QX4: fourth questionnaire
