# Supplementary material for: Childhood exposure to second-hand smoke (SHS) and risk of breast cancer in postmenopausal never smokers: the Multiethnic Cohort (MEC) study
Source: Breast Cancer Res. 2025 Dec 25;28:24. doi: 10.1186/s13058-025-02202-7 (PMC12849113; doi:10.1186/s13058-025-02202-7)
Supplement: Supplementary file 1 — Supplementary Material 1 [file 13058_2025_2202_MOESM1_ESM.docx]

Table S1. Age-standardized incidence rates (95% confidence intervals) of breast cancer by race and ethnicity

| Race/ethnicity | Total never smokers  (n=24,261) | | Second-hand smoke at home during childhood | | | |
| --- | --- | --- | --- | --- | --- | --- |
|  |  |  | No (n=14,751) | | Yes (n=9,509) | |
|  | No. of cases | Rates | No. of cases | Rates | No. of cases | Rates |
| African American | 83 | 436.8 (259.4-614.2) | 47 | 225.1 (151.1-299.1) | 36 | 669.9 (318.9-1020.9) |
| Japanese American | 287 | 386.8 (317.3-456.3) | 161 | 356.4 (261.7-451.2) | 126 | 422.9 (319.9-525.9) |
| Latino | 114 | 236.8 (158.9-314.6) | 74 | 219.8 (122.1-317.4) | 40 | 273.2 (142.5-403.9) |
| Native Hawaiian | 67 | 436.6 (318.4-554.8) | 40 | 451.4 (307.3-595.6) | 27 | 420.2 (226.1-614.2) |
| White | 158 | 287.6 (230.1-345.1) | 70 | 280.2 (194.3-366.2) | 88 | 296.4 (218.8-374.1) |

Rates (incidence/100,000) were adjusted to the 2000 US standard population and left truncated at age 60. Observation period is from

QX4 to the first event of invasive breast cancer diagnosis, death, or censor date of 2019.

Table S2. Second-hand smoke (SHS) at home during childhood and breast cancer risk by exposed hours per day and exposed years, the Multiethnic Cohort Study, 2008-2019

|  | Complete-case approach | | | All study population | | | |
| --- | --- | --- | --- | --- | --- | --- | --- |
| SHS exposure | No. of  participants | No. of  cases | HR (95% CI)^a^ | No. of  participants | No. of  cases | Separate category  for missing values | Imputed  missing values |
|  |  |  |  |  |  | HR (95% CI)^a^ | HR (95% CI)^a^ |
| No^b^ | 13297 | 366 | 1.00 (ref) | 14752 | 392 | 1.00 (ref) | 1.00 (ref) |
| Yes | 8785 | 295 | 1.10 (0.94-1.29) | 9509 | 317 | 1.12 (0.96-1.31) | 1.13 (0.97-1.31) |
| Among exposed |  |  |  |  |  |  |  |
| Exposed hours |  |  |  |  |  |  |  |
| <3 h/day | 3331 | 110 | 1.06 (0.85-1.32) | 3575 | 116 | 1.07 (0.87-1.32) | 1.07 (0.87-1.32) |
| ≥3 h/day | 3910 | 139 | 1.14 (0.93-1.40) | 4159 | 148 | 1.16 (0.95-1.42) | 1.17 (0.96-1.43) |
| P_trend_^c^ |  |  | 0.20 |  |  | 0.13 | 0.12 |
| Exposed years |  |  |  |  |  |  |  |
| <18 years | 4067 | 128 | 1.04 (0.85-1.28) | 4385 | 137 | 1.06 (0.87-1.29) | 1.06 (0.87-1.30) |
| ≥18 years | 3798 | 141 | 1.18 (0.97-1.45) | 4033 | 148 | 1.19 (0.98-1.45) | 1.20 (0.99-1.46) |
| P_trend_^c^ |  |  | 0.11 |  |  | 0.08 | 0.07 |
| Exposed hours and years |  |  |  |  |  |  |  |
| <3 h/day & <18 years | 1940 | 58 | 0.96 (0.73-1.28) | 2090 | 62 | 0.98 (0.75-1.29) | 0.98 (0.75-1.29) |
| ≥3 h/day & <18 years | 1611 | 54 | 1.10 (0.82-1.48) | 1710 | 57 | 1.11 (0.84-1.48) | 1.12 (0.84-1.49) |
| <3 h/day & ≥18 years | 1197 | 45 | 1.18 (0.86-1.62) | 1259 | 46 | 1.17 (0.86-1.60) | 1.18 (0.86-1.60) |
| ≥3 h/day & ≥18 years | 2151 | 80 | 1.17 (0.91-1.50) | 2274 | 85 | 1.19 (0.94-1.52) | 1.20 (0.94-1.53) |
| P_trend_^d^ |  |  | 0.13 |  |  | 0.09 | 0.09 |

^a^Adjusted for age at QX4, race/ethnicity, education, age at menarche, parity, number of children, BMI, physical activity, and alcohol drinker status.

^b^Common reference group.

^c^Trend variable assigned consecutive numbers for the three categories including the common reference group.

^d^Trend variable assigned consecutive numbers for the five categories including the common reference group.

Table S3. Second-hand smoke (SHS) at home during childhood and breast cancer risk, the Multiethnic Cohort Study, 2008-2019

| SHS  Exposure | ER-positive | | ER-negative | | PR-positive | | PR-negative | | HER2-postivie | | HER2-negative | |
| --- | --- | --- | --- | --- | --- | --- | --- | --- | --- | --- | --- | --- |
|  | No. of  cases | HR  (95% CI)^a^ | No. of  cases | HR  (95% CI)^a^ | No. of  cases | HR  (95% CI)^a^ | No. of  cases | HR  (95% CI)^a^ | No. of  cases | HR  (95% CI)^a^ | No. of  cases | HR  (95% CI)^a^ |
| Compete-case approach | | | | | | | | | | | | |
| No | 321 | 1.00  (ref) | 37 | 1.00  (ref) | 287 | 1.00  (ref) | 69 | 1.00  (ref) | 25 | 1.00  (ref) | 323 | 1.00  (ref) |
| Yes | 252 | 1.06  (0.89-1.26) | 36 | 1.40  (0.87-2.24) | 218 | 1.00  (0.84-1.20) | 69 | 1.49  (1.06-2.10) | 31 | 1.75  (1.02-3.02) | 252 | 1.05  (0.89-1.25) |
| P_heterogeneity_^b^ | 0.29 | | | | 0.06 | | | | 0.06 | | | |
| Separate category for missing values | | | | | | | | | | | | |
| No | 341 | 1.00  (ref) | 41 | 1.00  (ref) | 306 | 1.00  (ref) | 74 | 1.00  (ref) | 27 | 1.00  (ref) | 342 | 1.00  (ref) |
| Yes | 269 | 1.08  (0.92-1.27) | 40 | 1.45  (0.93-2.27) | 233 | 1.02  (0.86-1.22) | 75 | 1.54  (1.11-2.14) | 35 | 1.87  (1.12-3.14) | 269 | 1.08  (0.92-1.27) |
| P_heterogeneity_^b^ | 0.24 | | | | 0.04 | | | | 0.04 | | | |
| Imputed missing values | | | | | | | | | | | | |
| No | 341 | 1.00  (ref) | 41 | 1.00  (ref) | 306 | 1.00  (ref) | 74 | 1.00  (ref) | 27 | 1.00  (ref) | 342 | 1.00  (ref) |
| Yes | 269 | 1.08  (0.92-1.28) | 40 | 1.46  (0.93-2.29) | 233 | 1.03  (0.86-1.22) | 75 | 1.54  (1.11-2.15) | 35 | 1.88  (1.12-3.15) | 269 | 1.08  (0.92-1.28) |
| P_heterogeneity_^b^ | 0.24 | | | | 0.045 | | | | 0.04 | | | |

^a^Adjusted for age at QX4, race/ethnicity, education, age at menarche, parity, number of children, BMI, physical activity, and alcohol drinker status.

^b^Based on a competing risk model.

Table S4. Second-hand smoke (SHS) in childhood and adulthood and breast cancer risk, 2008-2019

| SHS exposure | No. of  participants | % | No. of  cases | HR (95% CI)^a^ |
| --- | --- | --- | --- | --- |
| Childhood + adulthood |  |  |  |  |
| No to both | 10602 | 43.7 | 276 | 1.00 (ref) |
| Yes to both | 5369 | 22.1 | 190 | 1.27 (1.06-1.54) |
| Yes to adulthood only | 2965 | 12.2 | 92 | 1.19 (0.94-1.51) |
| Missing | 1185 | 4.9 | 24 | 0.88 (0.58-1.35) |
| Childhood + adulthood + work |  |  |  |  |
| No to all | 7851 | 32.4 | 199 | 1.00 (ref) |
| Yes to all | 3928 | 16.2 | 146 | 1.34 (1.08-1.66) |
| Yes to adulthood or work only | 5700 | 23.5 | 170 | 1.15 (0.94-1.42) |
| Missing | 1201 | 5.0 | 23 | 0.84 (0.55-1.30) |
| Childhood + adulthood + work + current |  |  |  |  |
| No to all | 7679 | 31.7 | 195 | 1.00 (ref) |
| Yes to all | 1155 | 4.8 | 43 | 1.34 (0.96-1.86) |
| Yes to adulthood, work, or current only | 5823 | 24.0 | 172 | 1.14 (0.93-1.40) |
| Missing | 1250 | 5.2 | 25 | 0.89 (0.58-1.35) |

^a^Adjusted for age at QX4 and race/ethnicity.

Table S5. Selected characteristics of never smokers, overall and by exposure to second-hand smoke at home during childhood, excluding women aged 80 years or older, the Multiethnic Cohort Study

| Characteristics at cohort entry | Never smokers | Second-hand smoke exposure | | P-value^a^ |
| --- | --- | --- | --- | --- |
|  | Total | No | Yes |  |
| No. of participants | 17,253 | 9863 | 7390 |  |
| Age at QX4, mean (SD) | 70.2 (5.2) | 70.7 (5.2) | 69.5 (5.2) | <0.001 |
| Age at diagnosis, mean (SD) | 73.9 (5.5) | 74.4 (5.7) | 73.5 (5.3) | 0.07 |
| Years of follow-up, mean (SD) | 8.2 (1.8) | 8.2 (1.8) | 8.2 (1.8) | 0.21 |
| No. of breast cancer cases (%) | 544 (3.2) | 276 (2.8) | 268 (3.6) | 0.002 |
| Incidence/100,000 (95% CI)^b^ | 339.9 (294.4-385.4) | 294.3 (238.2-350.3) | 391.6 (319.4-463.7) |  |
| Race and ethnicity, n (%) |  |  |  |  |
| African American | 2013 (11.7) | 1241 (12.6) | 772 (10.4) | <0.001 |
| Japanese American | 5514 (32.0) | 3142 (31.9) | 2372 (32.1) |  |
| Latino | 4318 (25.0) | 3015 (30.6) | 1303 (17.6) |  |
| Native Hawaiian | 1200 (7.0) | 652 (6.6) | 548 (7.4) |  |
| White | 4208 (24.4) | 1813 (18.4) | 2395 (32.4) |  |
| Family history of breast cancer, n (%) | 1626 (9.8) | 892 (9.5) | 734 (10.2) | 0.10 |
| >12 years of education, n (%) | 11604 (67.8) | 6151 (63.0) | 5453 (74.3) | <0.001 |
| Age at menarche, mean (SD) | 13.0 (1.7) | 13.0 (1.7) | 12.9 (1.7) | <0.001 |
| Age at natural menopause, mean (SD) | 48.9 (4.8) | 49.0 (4.8) | 48.9 (4.8) | 0.36 |
| Parous women, n (%) | 14896 (86.5) | 8600 (87.4) | 6296 (85.3) | <0.001 |
| Number of children, mean (SD)^c^ | 3.0 (1.6) | 3.2 (1.6) | 2.9 (1.5) | <0.001 |
| Age at first childbirth, mean (SD)^c^ | 23.8 (4.6) | 23.7 (4.7) | 23.8 (4.6) | 0.15 |
| Body mass index at QX4 (kg/m^2^), mean (SD) | 26.5 (5.6) | 26.4 (5.5) | 26.8 (5.8) | <0.001 |
| Physical activity at QX4 (h/day), mean (SD)^d^ | 1.32 (1.33) | 1.30 (1.34) | 1.35 (1.33) | 0.02 |
| Nondrinkers at QX4, n (%) | 12010 (70.6) | 7353 (75.6) | 4657 (63.8) | <0.001 |

QX4, fourth questionnaire.

^a^T-test or chi-square test for differences between exposed and nonexposed groups.

^b^Rates were age-standardized to the 2000 US standard population and left truncated at age 60 years.

^c^Among parous women.

^d^Moderate to vigorous activities

Table S6. Second-hand smoke (SHS) at home during childhood and breast cancer risk by exposed hours per day and exposed years, excluding women aged 80 years or older, the Multiethnic Cohort Study, 2008-2019

| SHS exposure | No. of participants | No. of cases | HR (95% CI)^a^ |
| --- | --- | --- | --- |
| No^b^ | 9863 | 276 | 1.00 (ref) |
| Yes | 7390 | 268 | 1.24 (1.05-1.47) |
| Among exposed |  |  |  |
| Exposed hours |  |  |  |
| <3 h/day | 2748 | 105 | 1.26 (1.00-1.58) |
| ≥3 h/day | 3443 | 123 | 1.23 (0.98-1.53) |
| P_trend_^c^ |  |  | 0.0372 |
| Exposed years |  |  |  |
| <18 years | 3452 | 117 | 1.17 (0.94-1.45) |
| ≥18 years | 3219 | 128 | 1.33 (1.07-1.65) |
| P_trend_^c^ |  |  | 0.0073 |
| Exposed hours and years |  |  |  |
| <3 h/day & <18 years | 1624 | 58 | 1.19 (0.89-1.58) |
| ≥3 h/day & <18 years | 1424 | 47 | 1.16 (0.84-1.58) |
| <3 h/day & ≥18 years | 965 | 41 | 1.38 (0.99-1.92) |
| ≥3 h/day & ≥18 years | 1902 | 73 | 1.29 (0.99-1.69) |
| P_trend_^d^ |  |  | 0.0175 |

^a^Adjusted for age at QX4 and race/ethnicity.

^b^Common reference group.

^c^Trend variable assigned consecutive numbers for the three categories including the common reference group.

^d^Trend variable assigned consecutive numbers for the five categories including the common reference group.

Table S7. Second-hand smoke (SHS) at home during childhood and breast cancer risk, excluding women aged 80 years or older, the Multiethnic Cohort Study, 2008-2019

| SHS  Exposure | ER-positive | | ER-negative | | PR-positive | | PR-negative | | HER2-postivie | | HER2-negative | |
| --- | --- | --- | --- | --- | --- | --- | --- | --- | --- | --- | --- | --- |
|  | No. of  cases | HR  (95% CI)^a^ | No. of  cases | HR  (95% CI)^a^ | No. of  cases | HR  (95% CI)^a^ | No. of  cases | HR  (95% CI)^a^ | No. of  cases | HR  (95% CI)^a^ | No. of  cases | HR  (95% CI)^a^ |
| No | 245 | 1.00  (ref) | 28 | 1.00  (ref) | 225 | 1.00  (ref) | 48 | 1.00  (ref) | 16 | 1.00  (ref) | 246 | 1.00  (ref) |
| Yes | 232 | 1.20  (1.00-1.44) | 31 | 1.51  (0.90-2.54) | 202 | 1.12  (0.92-1.36) | 60 | 1.71  (1.16-2.52) | 31 | 2.57  (1.39-4.76) | 227 | 1.16  (0.97-1.40) |
| P_heterogeneity_^b^ | 0.41 | | | | 0.06 | | | | 0.01 | | | |

^a^Adjusted for age at QX4 and race/ethnicity.

^b^Based on a competing risk model.

Table S8. Second-hand smoke (SHS) at home during childhood and breast cancer risk by nine risk factor subgroups, excluding women aged 80 years or older, the Multiethnic Cohort Study, 2008-2019

| Subgroup | SHS  exposure | No. of  participants | No. of  cases | HR (95% CI)^a^ | P_heterogeneity_ |
| --- | --- | --- | --- | --- | --- |
| Year of birth |  |  |  |  |  |
| 1930-1939 | No | 4960 | 142 | 1.00 (ref) |  |
|  | Yes | 2899 | 96 | 1.11 (0.86-1.45) |  |
| ≥1940 | No | 4903 | 134 | 1.00 (ref) |  |
|  | Yes | 4491 | 172 | 1.36 (1.08-1.71) | 0.19 |
| Age at QX4 |  |  |  |  |  |
| 60-69 years | No | 4170 | 120 | 1.00 (ref) |  |
|  | Yes | 3921 | 154 | 1.34 (1.06-1.71) |  |
| 70-79 years | No | 5693 | 156 | 1.00 (ref) |  |
|  | Yes | 3469 | 114 | 1.15 (0.90-1.47) | 0.34 |
| Race and ethnicity |  |  |  |  |  |
| African American | No | 1241 | 31 | 1.00 (ref) |  |
|  | Yes | 772 | 33 | 1.69 (1.03-2.77) |  |
| Japanese American | No | 3142 | 101 | 1.00 (ref) |  |
|  | Yes | 2372 | 107 | 1.39 (1.06-1.83) |  |
| Latino | No | 3015 | 55 | 1.00 (ref) |  |
|  | Yes | 1303 | 34 | 1.41 (0.92-2.16) |  |
| Native Hawaiian | No | 652 | 35 | 1.00 (ref) |  |
|  | Yes | 548 | 23 | 0.79 (0.46-1.34) |  |
| White | No | 1813 | 54 | 1.00 (ref) |  |
|  | Yes | 2395 | 71 | 1.00 (0.70-1.42) | 0.12 |
| Education |  |  |  |  |  |
| ≤12 years | No | 3609 | 72 | 1.00 (ref) |  |
|  | Yes | 1891 | 52 | 1.24 (0.86-1.79) |  |
| >12 years | No | 6151 | 202 | 1.00 (ref) |  |
|  | Yes | 5453 | 213 | 1.20 (0.99-1.46) | 0.71 |
| Age at menarche |  |  |  |  |  |
| ≤12 years | No | 5068 | 165 | 1.00 (ref) |  |
|  | Yes | 4138 | 158 | 1.15 (0.92-1.44) |  |
| >12 years | No | 4694 | 108 | 1.00 (ref) |  |
|  | Yes | 3184 | 108 | 1.37 (1.04-1.80) | 0.20 |
| Parity |  |  |  |  |  |
| Nulliparous | No | 1236 | 37 | 1.00 (ref) |  |
|  | Yes | 1081 | 42 | 1.36 (0.86-2.13) |  |
| Parous | No | 8600 | 239 | 1.00 (ref) |  |
|  | Yes | 6296 | 225 | 1.22 (1.01-1.47) | 0.84 |
| Body mass index at QX4 |  |  |  |  |  |
| <25 kg/m^2^ | No | 4325 | 100 | 1.00 (ref) |  |
|  | Yes | 3111 | 97 | 1.32 (1.00-1.76) |  |
| ≥25 kg/m^2^ | No | 5044 | 167 | 1.00 (ref) |  |
|  | Yes | 3987 | 163 | 1.15 (0.92-1.43) | 0.45 |
| Physical activity at QX4 |  |  |  |  |  |
| <45 min/day | No | 4350 | 110 | 1.00 (ref) |  |
|  | Yes | 3043 | 102 | 1.24 (0.94-1.63) |  |
| ≥45 min/day | No | 5357 | 164 | 1.00 (ref) |  |
|  | Yes | 4266 | 165 | 1.25 (1.00-1.56) | 0.95 |
| Alcohol consumption at QX4 |  |  |  |  |  |
| Nondrinkers | No | 7353 | 203 | 1.00 (ref) |  |
|  | Yes | 4657 | 172 | 1.25 (1.02-1.54) |  |
| Drinkers | No | 2372 | 71 | 1.00 (ref) |  |
|  | Yes | 2639 | 96 | 1.25 (0.92-1.71) | 0.83 |

QX4, fourth questionnaire.

^a^Adjusted for age at QX4 and race/ethnicity.
